# Supplementary material for: Smooth Interpolating Curves with Local Control and Monotone Alternating Curvature
Source: Comput Graph Forum. 2022 Oct 6;41(5):25–38. doi: 10.1111/cgf.14600 (PMC9827861; doi:10.1111/cgf.14600)
Supplement: Supplementary file 1 — Supplement Material [file CGF-41-25-s001.zip › Local-Smooth-Interpolating-MonoCurvature/extern/clothoids/submodules/Utils/docs/api-cpp/file_Utils_ThreadPool.hxx.html]

File ThreadPool.hxx — Utils v1.2

### Navigation

- index
- toc
- Utils »
- File ThreadPool.hxx

# File ThreadPool.hxx¶

↰ Parent directory (`Utils`)

Contents

- Definition (`Utils/ThreadPool.hxx`)

## Definition (`Utils/ThreadPool.hxx`)¶

- Program Listing for File ThreadPool.hxx

### Quick search

### Table of Contents

- rang - Colors for your Terminal
- Zstr
- C++ API

«
hide menu

menu
sidebar
»

### Navigation

- index
- toc
- Utils »
- File ThreadPool.hxx

© Copyright 2021, Enrico Bertolazzi.
Created using Sphinx 4.2.0.
